# Neuropsychomotor Development of Children Exposed to SARS-CoV-2 in Utero During COVID-19 Pandemic

**DOI:** 10.3390/biomedicines13092256

**Published:** 2025-09-12

**Authors:** Felipe Motta, Maria Eduarda Canellas-de-Castro, Geraldo Magela Fernandes, Lizandra Moura Paravidine Sasaki, David Alves de Araújo Júnior, Alberto Moreno Zaconeta, Ângelo Pereira da Silva, Ciro Martins Gomes, Cleandro Pires Albuquerque, Ismael Artur Costa-Rocha, Janaina Araújo Teixeira Santos, José Alfredo Lacerda De Jesus, Karina Nascimento Costa, Laila Salmen Espindola, Licia Maria Henrique da Mota, Lucas Lauand, Luiz Cláudio Gonçalves de Castro, Marcelo Antônio Pascoal Xavier, Jordana Grazziela Alves Coelho-dos-Reis, Otávio Toledo Nóbrega, Pabline Cavalcante da Silva, Rodrigo de Resende Nery, Wanessa Tavares Santos, Rosana Maria Tristão, Caroline Oliveira Alves, Olindo Assis Martins-Filho, Alexandre Anderson de Sousa Munhoz Soares

**Affiliations:** 1 Programa de Pós-Graduação em Ciências Médicas, Universidade de Brasília (UnB), Brasília 70910-900, Distrito Federal, Brazil; maria.castro@unb.br (M.E.C.-d.-C.); geraldomafer@gmail.com (G.M.F.); lizandra78@gmail.com (L.M.P.S.); juniorfish@gmail.com (D.A.d.A.J.); ciromgomes@gmail.com (C.M.G.); cleandropires@hotmail.com (C.P.A.); darvenne@gmail.com (L.S.E.); liciamhmota@gmail.com (L.M.H.d.M.); lc-castro@uol.com.br (L.C.G.d.C.); otavionobrega@unb.br (O.T.N.); 2 Hospital Universitário de Brasília, Universidade de Brasília (UnB), Brasília 70830-200, Distrito Federal, Brazil; angelopereiradasilva@yahoo.com.br; 3 Faculdade de Medicina, Universidade de Brasilia (UnB), Brasília 70910-900, Distrito Federal, Brazil; azaconeta@gmail.com (A.M.Z.); jalfredo.jesus@gmail.com (J.A.L.D.J.); karinacosta@unb.br (K.N.C.); lucas.lauand@gmail.com (L.L.); rodrigo6749@gmail.com (R.d.R.N.); rosana.tristao@gmail.com (R.M.T.); 4 Programa de Pós-Graduação em Patologia Molecular, Universidade de Brasilia (UnB), Brasília 70910-900, Distrito Federal, Brazil; 5 Instituto René Rachou, Fundação Oswaldo Cruz (FIOCRUZ-Minas), Belo Horizonte 30190-002, Minas Gerais, Brazil; ismaelacrocha@gmail.com (I.A.C.-R.); mpascoal@ufmg.br (M.A.P.X.); oamfilho@gmail.com (O.A.M.-F.); 6 Secretaria de Saúde do Distrito Federal, Brasília 70719-040, Distrito Federal, Brazil; janafisiot@gmail.com; 7 Faculdade de Medicina, Universidade Federal de Minas Gerais, Belo Horizonte 30130-100, Minas Gerais, Brazil; 8 Instituto de Ciências Biológicas, Universidade Federal de Minas Gerais, Belo Horizonte 30130-100, Minas Gerais, Brazil; jreis@icb.ufmg.br; 9 Faculdade de Terapia Ocupacional, Universidade de Brasilia (UnB), Ceilândia 72220-275, Distrito Federal, Brazil; pabline.cavalcante@gmail.com (P.C.d.S.); wanessatavares73@gmail.com (W.T.S.); carolineoliveiraalves@gmail.com (C.O.A.)

**Keywords:** neonatal disease, child development, COVID-19, pandemic, SARS-CoV-2, neuropsychological tests, infants

## Abstract

**Introduction**: Little is known about the effects of intrauterine exposure to SARS-CoV-2, especially on growth and neurodevelopment in children. **Objective**: We wished to verify the effect of intrauterine exposure to SARS-CoV-2 on neurological development in children. **Methods**: Infants born to mothers presenting with SARS-CoV-2 infection during pregnancy were enrolled in a prospective descriptive–analytical study involving outpatient appointments performed 6 and 12 months after birth. Their neurological development was assessed using the Bayley-III Scale, using a score of >85 as the cutoff threshold for identifying developmental delay. Differences between groups were assessed through an ANOVA, using Bonferroni correction for multiple comparisons. Regression models were employed to examine the associations between the Bayley-III scores and maternal features. **Results**: Two hundred and sixty-nine infants were evaluated, most of whom were born full-term and with birth weights appropriate for gestational age at delivery. Developmental delays were observed in 26% of the infants in at least one of the Bayley-III domains. The language domain was particularly affected, with impairments observed in children exposed to SARS-CoV-2 closer to the time of delivery. These findings were statistically significant (*p* < 0.05). **Conclusions**: Infants born to mothers presenting with SARS-CoV-2 infection during pregnancy presented developmental delays at 6 and 12 months, particularly in the language domain. These findings reinforce the relevance of long-term clinical follow-ups of newborns exposed to SARS-CoV-2 infection during pregnancy.

## 1. Introduction

Neuropsychomotor developmental delays in children exposed to SARS-CoV-2 in utero during the COVID-19 pandemic have been suggested, particularly in the language and motor domains. Previous research has been conducted to understand the relationship between maternal complications and SARS-CoV-2 infection during pregnancy [1]. However, the perinatal repercussions caused by SARS-CoV-2 have not yet been elucidated. There are gaps in the understanding of this process, which still requires more comprehensive elucidation, especially regarding the impact of maternal SARS-CoV-2 infections on fetal and neonatal neurological and psychiatric development [2,3].

It has been shown that maternal infections during pregnancy, followed by congenital fetal infections, can affect fetal development through direct infections in fetal cells. Exposure of the fetus to maternal inflammation is associated with placental damage, which may compromise fetal development. Several congenital infections, such as Cytomegalovirus, Zika, and Rubella viral infections, as well as Syphilis and Toxoplasmosis, have been associated with neurodevelopmental impairments [4]. Although children exposed to infectious agents during pregnancy may not present symptoms at birth, they may display neurodevelopmental commitment over time, with the sequelae varying according to the pathogen and the severity of the infection. Previous research on neurodevelopment in infants born with congenital infections suggests that even without brain damage or birth defects, children may experience impaired neurodevelopment. This process could be associated with the inflammatory cytokine response and placental immune activation, which can interfere with brain growth and plasticity [5,6].

As maternal SARS-CoV-2 infections during pregnancy lead to an acute inflammatory status, they can impact fetal development, resulting in impaired neurological features. The current state of the knowledge provides evidence that SARS-CoV-2 exposure during pregnancy is associated with fetal neurodevelopmental impairments, suggesting that specific guidelines are needed to monitor and mitigate its long-term effects on children’s health [7]. Some studies have suggested that SARS-CoV-2 infections during pregnancy do not appear to be associated with early childhood developmental concerns [8]. Recent reports from our group argue against this point by demonstrating pediatric structural encephalic changes associated with SARS-CoV-2 infection during pregnancy [9]. As some developmental commitments are undiagnosed or inapparent until later in infancy, clinical follow-up should continue over time after birth. In this sense, the constant monitoring of neurodevelopment is essential during the first two years, a period when developmental milestones and neurocognitive acquisitions occur most significantly. Longitudinal studies have demonstrated that newborns exposed to SARS-CoV-2 prenatally had lower neurodevelopmental scores, particularly in motor development and interactive behavior [10]. Furthermore, Suffrey et al. [11] found that children born during the pandemic had lower-than-average scores in developmental tests. As suggested previously [2,10], further research is warranted to elucidate the full extent of these effects.

The present work was designed as a prospective cohort investigation aiming at providing evidence of the impact of in utero SARS-CoV-2 infection on infant neurological development. This study comprises two major goals, as follows: (i) examine comprehensively the neurological development of infants exposed in utero to SARS-CoV-2 during a 1-year follow-up and (ii) compare the development of the infants according to clinical aspects of SARS-CoV-2 exposure during pregnancy and the maternal education level. The findings of this study will contribute significantly to our understanding of the effects of prenatal exposure to SARS-CoV-2 on infant neurodevelopment as well as to clinical pediatric practice and public health policies.

## 2. Material and Methods

### 2.1. Population

This is a prospective cohort study performed at the University Hospital of Brasília, in Brasília, Brazil. Children were eligible for enrollment from the first 15 days of life to 12 months of age if born from June 2020 to November 2020 and if their mothers had had SARS-CoV-2 infection proven by nasopharyngeal RT-PCR during pregnancy. The primary outcome was evaluated with the Bayley Scales of Infant and Toddler Development, Third Edition (Bayley-III) at 6 and 12 months of life. The participants were recruited with non-probabilistic convenience sampling, using media and social networks from 1 May 2020 to 31 May 2021. Infants enrolled in the study were followed prospectively from 1 July 2020 to 31 December 2021. This study was performed at two public hospitals—the University Hospital of Brasília and Asa Norte Regional Hospital, both considered public reference centers for COVID-19 in the Federal District of Brazil.

The exclusion criteria applied in this investigation comprised pregnant women vaccinated for COVID-19 before or during pregnancy. In addition, smokers and excessive alcohol/illicit drug users were excluded. The exclusion criteria also encompassed neonatal features, including requirement of Intensive Care Unit admission, congenital malformations, and chromosomal abnormalities, as well as clinical and/or serological diagnoses of congenital infections (Cytomegalovirus, Zika, Rubella, Hepatitis B and C viral infection, Chagas disease, Syphilis, or Toxoplasmosis). To reduce the risk of confounding bias for the clinical features and outcomes, pregnant women with a confirmed diagnosis of Cytomegalovirus, Zika, Rubella, Herpes, Human Immunodeficiency Virus infection, Chagas disease, Syphilis, or Toxoplasmosis were excluded from the study. Upon enrollment, no children were excluded from the study population.

All participants underwent mandatory neonatal screenings as per Brazilian Health Ministry guidelines, including biological screening (TANDEM methodology), pulse oximetry test with subsequent echocardiogram if abnormalities were detected, auditory screening (evoked otoacoustic emissions), and ophthalmic fundus examination.

The evaluation took place with pregnant women infected by SARS-CoV-2 in Brazil between June 2020 and April 2022. The guardians of each participant signed an informed consent form and were informed of the test results. Children with developmental delays were advised and referred to a rehabilitation center.

**Figure 1:**
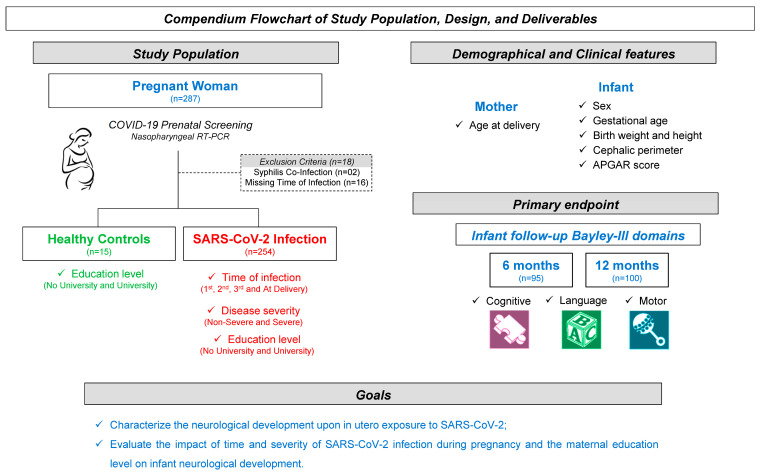
Compendium flowchart of study design, population, and methods.

Figure 1 presents a compendium flowchart of study design, population, and methods.

### 2.2. Data Collection Procedures, Instruments, and Analyzed Variables

A detailed study description has been previously published [3]. In this manuscript, we provide a summary of the overall methodology employed in the study. Information of the clinical status of the pregnant women was collected and analyzed during initial consultations, whereas data on infants and children were gathered during follow-up visits. Maternal infection severity was graded as non-severe, severe, and critical according to the criteria of the World Health Organization (WHO), and pregnant women were divided into 2 groups, non-severe and severe (including critical) groups, for analysis purposes. The trimester of infection was defined as follows: 1st trimester (up to 13 weeks and 6 days), 2nd trimester (from 14 weeks to 26 weeks and 6 days), and 3rd trimester (more than 27 weeks). Acute infection at delivery was considered a separate clinical subgroup. The caregiver educational level was determined according to the presence or absence of a university degree.

The Bayley-III Scale is the most used assessment tool for infant neurodevelopment [12]. The original version presents appropriate parameters of validity and reliability, in addition to good sensitivity and specificity indices to identify children with developmental delay [13,14,15]. These scales were translated into Portuguese and validated in the Brazilian context in 2006, and the Bayley-III Scale has become the gold standard for monitoring children’s developmental progress since then [13,14]. The Bayley-III Scales were applied by four independent examiners, previously trained by professionals from the Occupational Therapy Department at the University of Brasília, with at least 10 years of experience with Bayley Scales application to ensure the accuracy and consistency of data collection. In this study, the Bayley-III Scales were used to assess infant neurodevelopment, focusing on the motor, cognitive, and language subscales, while the complementary scales (socio-emotional and adaptive behavior) were not utilized. The original version of Bayley-III demonstrates adequate validity and reliability parameters, with strong sensitivity and specificity indices for identifying children with developmental delays [10,11]. Furthermore, as an additional external control, inter-rater reliability was applied by performing measurements in 10 children who were not part of the present study, resulting in an excellent correlation index (ICC = 90).

To evaluate the performance of the investigated children, a score of 0 or 1 was attributed to each item on the scale. The weighted average of the scores obtained by the Bayley-III at 6 and 12 months was used. The Bayley scores were expressed as continuous variables and converted into categorical data, considering the cutoff threshold of <85 to indicate an altered score, considered a delay in neurological development [16,17].

### 2.3. Statistical Analysis

Normality tests (Shapiro–Wilk and Kolmogorov–Smirnov) were carried out to determine the dataset distribution profile. The clinical and demographic parameters were presented as means (± standard deviations—SDs) and as frequency values. Multiple comparisons amongst groups were performed using the analysis of variance (ANOVA) test with Bonferroni correction. Comparative analyses of categorical data were carried out by the Chi-square test (χ^2^). Multinomial and logistic regression models were employed, adjusting for infant sex and gestational age at birth to verify associations of the cognitive, language, and motor domains of the Bayley-III Scale with maternal features (time of SARS-CoV-2 infection, disease severity, and educational level). The statistical analyses were performed using Statistical Analysis System software (SAS; version 9.4), Graphpad Prism Software (version 10.0), and Minitab software (Minitab, LLC; version 17). In all cases, *p*-values < 0.05 were considered statistically significant.

## 3. Results

### 3.1. Characterization of Study Population

Table 1 summarizes the clinical and demographic features of the study population. Most babies were born full-term (90.3%), and the mean gestational age at birth was 38.40 (±1.66) weeks. Around half of the participants were females (51.3%) with a mean birth weight of 3137.80 (±532.84) grams. Most babies (80.1%) had an appropriate weight for their gestational age at birth. The mean birth height was 51.46 (±6.69) cm. The cephalic perimeter was 35.50 (±3.62) cm.

The mother mean age at delivery was 30.52 (±6.34) years. The time of SARS-CoV-2 infection during pregnancy comprised 19.1% in the first trimester, 34.5% in the second trimester, 38.9% in the third trimester, and 7.5% with acute infection at delivery. Most mothers presented non-severe illness (84.6%), with 15.4% presenting severe disease. The caregiver educational level indicated that a high proportion of mothers (72.6%) did not have a university degree (Table 1).

### 3.2. Bayley-III Developmental Delays Regardless of Unaltered APGAR Scores

**Figure 2:**
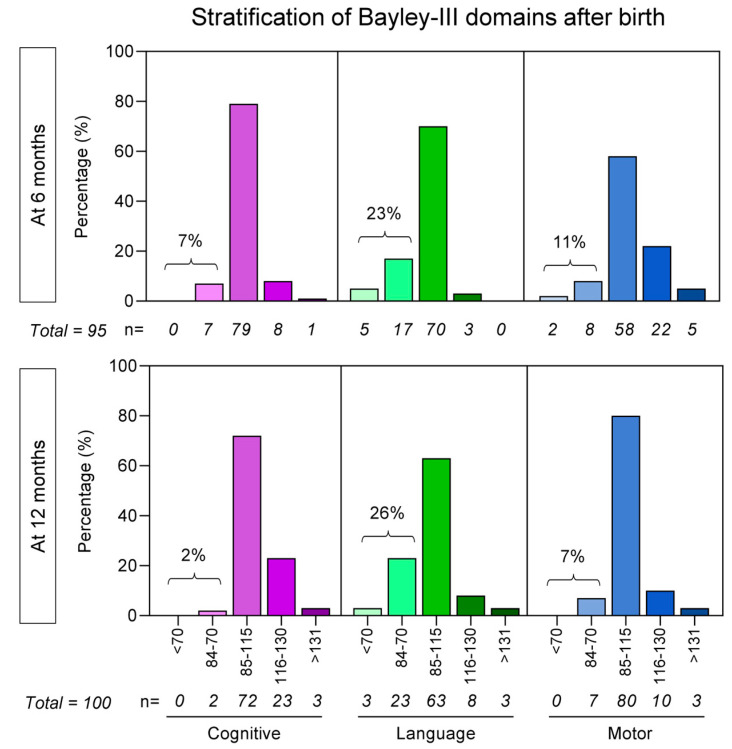
Stratification of Bayley-III cognitive (purple bars), language (green bars), and motor (blue bars) domains at 6 and 12 months after birth for infants born to mothers with SARS-CoV-2 infection during pregnancy. Data are presented as percentages of children with Bayley-III categorized as  <70 (<−2 SD below average), 84–70 (−1 to −2 SD below average), 85–115 (−1 to 1 SD around average), 116–130 (1–2 SD above average), and  >131 (>2 SD above average), illustrated by distinct shades of colors. SD = standard deviation.

The categorical analysis of the Bayley-III scores to assess the infant neurological development status at 6 and 12 months of age is presented in Figure 2. The results indicate that at 6 months of age, 7% of the infants born to mothers infected with SARS-CoV-2 during pregnancy experienced cognitive delays, 23% had language delays, and 11% had motor delays. As for 12 months of age, cognitive delays were observed in 2% of the participants, language delays in 26%, and motor delays in 7%. The percentage of children with a delay according to each group can be observed in Figure 2.

No significant differences were observed in the frequency of the APGAR 1st- and 5th-minute scores (*p* = 0.190 and 0.4695, respectively) between infants born to mothers infected with SARS-CoV-2 during pregnancy (≤7–20.7% and 4.1%, respectively) and healthy controls (≤7–35.7% and 7.1%, respectively). Therefore, there is no evidence suggesting an association between APGAR scores and cognitive, language, and motor development (Table 1).

### 3.3. Bayley-III Domains According to the Time of Maternal SARS-CoV-2 Infection

Table 2 presents the comparative analysis between cognitive, language, and motor domains of infants at 6 and 12 months, according to the trimester during pregnancy in which maternal SARS-CoV-2 infection occurred. The data analysis demonstrated that at 6 months, a lower cognitive domain was observed for infants born to mothers who acquired SARS-CoV-2 infection in the third trimester or with acute infection at delivery compared with those infected in the first pregnancy trimester.

At 12 months, lower language development was observed in infants born to mothers with acute infection at delivery compared with those born to mothers infected in the first pregnancy trimester.

The analysis of the categorical Bayley-III scores demonstrated that except for the cognitive domain at 12 months, most Bayley-III scores presented altered patterns in infant born to mothers infected with SARS-CoV-2 during pregnancy compared with healthy controls.

Of note, infants born to mothers with acute infection at delivery presented a significant higher frequency of altered Bayley-III scores in most domains compared with those born to mothers infected earlier during pregnancy, especially in the first trimester (Table 2).

### 3.4. Bayley-III Domains According to the Severity of Maternal SARS-CoV-2 Infection

The Bayley-III cognitive, language, and motor domains at 6 and 12 months were also evaluated according to the severity of maternal SARS-CoV-2 infection during pregnancy (Table 3). The data analysis carried out by taking the dataset as continuous variables did not demonstrate significant differences in the Bayley-III domains at 6 and 12 months.

However, the analysis of the categorical Bayley-III scores at 6 and 12 months demonstrated that regardless of the disease severity, infants born to mothers with SARS-CoV-2 infection presented altered Bayley-III scores compared with those born to healthy control mothers.

Additionally, infants born to mothers with severe SARS-CoV-2 infection presented a higher frequency of altered language and motor domains at 6 months compared with the group with non-severe maternal disease. At 12 months, the frequency of infants with altered language and motor domains were lower in the subgroup born to mothers with severe SARS-CoV-2 infection (Table 3).

### 3.5. Bayley-III Domains According to the Caregiver Educational Level

Table 4 shows the comparison between the cognitive, language, and motor domains at 6 and 12 months according to the caregiver educational levels. The data analysis carried out by taking the dataset as continuous variables did not show significant differences in the Bayley-III domains at 6 and 12 months.

The analysis of the categorical data showed that higher frequencies of altered Bayley-III domains were found in infants born to mothers with SARS-CoV-2 infection during pregnancy compared with healthy controls. However, no differences in the categorical data were found regarding the caregiver educational level (Table 4).

### 3.6. Regression Models of Association Between Bayley-III Domains and Maternal Features

Multinomial and logistic regression models were employed to examine the associations between the Bayley-III scores of each domain and maternal features including time of infection, disease severity, and educational level (Table 5). The data analysis indicated a significant negative relationship between altered cognitive (coef = −14.5) and language (coef = −14.01) domains at 12 months and acute SARS-CoV-2 infection at delivery (Table 5).

## 4. Discussion

This study observed that women exposed to SARS-CoV-2 during pregnancy had children with delayed neuropsychomotor development, specifically in the language domain. For this, the individuals studied (all within the context of a pandemic and social isolation) were divided according to the trimester of infection onset, the severity of the infection in the pregnant woman, and the educational level of the caregivers. The analysis of the Bayley-III domains based on categorical classification demonstrated that at 6 months of life, delays in cognitive, language, and motor development were observed in infants born to SARS-CoV-2-infected mothers, especially in those from subgroups of mothers with acute infection at delivery, with severe COVID-19, and without a university degree. At 12 months, a language delay was observed particularly in children whose pregnant mothers were acutely infected with SARS-CoV-2 at delivery. Multinomial and logistic regression models indicated a significant negative relationship between altered cognitive and language domains at 12 months and acute SARS-CoV-2 infection at delivery.

Given these findings, once the SARS-CoV-2 infection has been diagnosed in a pregnant woman, an orderly and serial follow-up of these children is necessary, with a strong focus on developmental assessment for diagnosis and for promoting early intervention in order to reduce the impact of this delay on the child’s further development, since language acts as a driver of many other skills to be acquired in childhood and adult life [17].

In two recent studies, developmental tests were administered indirectly by asking parents to complete surveys. Despite observing differences in developmental scores between infants exposed to SARS-CoV-2 and those not exposed, these studies did not find any statistically significant differences between the groups according to the trimester of infection onset during pregnancy or the severity of the maternal disease [6,18]. However, children born during the pandemic exhibited a considerable reduction in verbal, motor, and general cognitive performance compared with those born prior to the pandemic, regardless of their exposure to the virus [18].

The mitigation of the effect of viral infection during pregnancy on speech was observed with the increase in age in clinical studies, which could be explained by routine pediatric interventions in response to any developmental delay. The early identification of language delays allows for targeted stimulation, parental guidance, and when necessary, speech therapy or other developmental interventions. These strategies help alleviate the initial impact of viral exposure on neurodevelopment, promoting language acquisition over time. Additionally, neural plasticity in early childhood may play a role in compensating for initial delays, as children have an inherent capacity to reorganize and strengthen alternative neural pathways in response to early-life challenges.

Furthermore, a recent survey has uncovered a concerning increase in pre-existing social disparities during the pandemic. Specifically, the survey, which interviewed 500 parents from disadvantaged backgrounds in terms of education, revealed that their 0-to-2-year-old children were less likely to participate in enriching activities. This social deprivation in engaging in activities may contribute to developmental delays, as was observed in the current study, particularly among children whose mothers did not hold a university degree [19,20]. These findings highlight the need for greater support and resources to be made available to vulnerable families during times of crisis [21,22].

Shuffrey et al. revealed a difference in gross and fine motor development and personal/social scores in pandemic babies compared with those born before the pandemic. These authors did not observe associations between in utero exposure to maternal SARS-CoV-2 infection and neurodevelopmental delay at 6 months of age. These results differ from our data, which show an impact of maternal SARS-CoV-2 infection during pregnancy and delayed neurological development at 6 and 12 months of age. Together, these findings support the need for the long-term monitoring of children born during the SARS-CoV-2 pandemic to assess putative late outcomes [11].

A previous study by our group was pioneering in utilizing both the gold-standard neurodevelopmental assessment scale and ultrasound elastography for the purpose of investigating possible impacts of the pandemic on children [9]. In this previous study, Junior and colleagues presented elastography data from children exposed and not exposed to SARS-CoV-2 during the pandemic, which showed statistically significant differences, particularly a reduction in the elasticity coefficient in the deep white matter in the exposed group. Notably, children with altered elasticity coefficients, possibly with prior inflammation, also exhibited developmental delays in the language subdomain of the Bayley-III Scale at 12 months. A plausible neurological model to explain these events is that viral exposure may lead to changes in myelin content or induce mild myelin edema. Inflammatory processes could increase water content in the brain parenchyma, altering its stiffness and reducing elasticity coefficients. Since myelin is essential to efficient neural transmission, these structural changes might disrupt connectivity within language-related brain networks, ultimately contributing to the observed language delays.

The present study presents key methodological differences compared with previous investigations. Unlike other studies that incorporated parental impressions in developmental evaluations, the current investigation has employed the Bayley-III Scale applied by experienced examiners who had no emotional bond or acquaintance with the child prior to the test. This approach minimizes potential biases, such as the overestimation of developmental outcomes due to parental perception. However, it is important to mention that this study has some limitations. The primary limitations include the relatively small sample size. In this sense, it is also important to point out that correlational studies do not establish cause–effect principles. Given that data collection began during the COVID-19 pandemic—during a period of social isolation and prior to vaccine availability—the population’s fear of attending a clinic located within a hospital complex (and the potential exposure to asymptomatic carriers, particularly via public transportation) could have impacted the recruitment of a larger control group. The inclusion of a pre-pandemic control group was inadequate, since the presence or absence of social isolation could introduce confounding variables, potentially affecting developmental outcomes. Additionally, the study population sample consisted of outpatients, meaning that maternal infections were predominantly mild. Furthermore, this study was conducted in a single center, which may limit the generalizability of our findings. Future studies with larger sample sizes and multicenter designs are necessary to validate and expand upon our results. Another limitation of our study is the number of maternal socio-demographic features assessed during the questionnaire application. The analysis of other maternal features such as income level, access to healthcare, social support, family composition, number of family members, the birth order of the children, and previous motherhood experience should be included in future investigations, since they may have a direct impact on child development. Possible interventions for these children and the impact of readjusting neurodevelopment scores, especially language, should also be monitored.

The research aims outlined in this study encompassed a comprehensive examination of the developmental trajectory in children exposed to SARS-CoV-2 in utero using the Bayley-III over a one-year period, as well as a comparative analysis based on factors such as the timing of fetal exposure, maternal infection severity, and maternal educational level. It is imperative to pursue further investigations into developmental delays and to understand the right moment for intervention as well as whether the intervention could change the course of the delay. Additionally, the evaluation of children’s development within a cohort of vaccinated pregnant women becomes paramount, as vaccinated individuals demonstrate distinctive responses to SARS-CoV-2 infection and associated conditions. This evaluation will enable an assessment of whether such children are also susceptible to developmental delays as in the case of unvaccinated mothers.

In conclusion, our findings indicate that children born to mothers exposed to SARS-CoV-2 during the COVID-19 pandemic exhibited delays in neuropsychomotor development, particularly in the language domain. This association was most evident in children whose mothers were infected at the time of delivery and in those with severe infections. Additionally, we observed that children whose caregivers did not have a university degree, which is considered a high educational level for the Brazilian population, were more likely to experience language developmental delays, suggesting that socioeconomic disparities may have played a significant role in shaping developmental outcomes during the pandemic. This finding underscores the need to consider environmental and social factors when evaluating the potential effects of in utero viral exposure. Given these results, we emphasize the importance of continuous follow-up and early intervention for children exposed to SARS-CoV-2 during gestation. Targeted screening and developmental support strategies may help mitigate potential long-term consequences, particularly in language acquisition, which is fundamental to cognitive development, socialization, and future academic achievement. Our findings also open new avenues for research into the effects of maternal infection on infant neurodevelopment. The longitudinal follow-up of these children could provide valuable insights into the consequences of in utero exposure to SARS-CoV-2 and its impact on the central nervous system. The observed association between SARS-CoV-2 exposure and altered neurodevelopment highlights the need for healthcare professionals to be vigilant in monitoring children born to mothers infected during pregnancy. Early screening and timely referral to appropriate interventions could mitigate developmental deficits and promote more favorable long-term outcomes. 

Finally, our results suggest that public health policies aimed at reducing disparities in access to healthcare and parental education may be crucial to minimizing the adverse effects of the pandemic on early childhood development. Building on our findings, we propose the following topics for future research: (i) the recruitment of patients from existing databases for subsequent Bayley-III assessments at 24 months of age to evaluate the trajectory of language developmental delays; (ii) advanced neuroimaging studies using brain MRI in children with developmental delays beyond 24 months, post-myelination, to identify potential structural and functional alterations associated with in utero viral exposure; and (iii) comparative studies on neurodevelopmental outcomes in children exposed to SARS-CoV-2 in utero, differentiating between those born to vaccinated and unvaccinated mothers, given the potential influence of maternal immune responses on fetal neurodevelopment.

## Figures and Tables

**Table 1:** Study population.

Clinical and Demographic Parameters	Healthy Controls * (*n* = 15)	SARS-CoV-2 Infection (*n* = 254)
Full term, *n* (%)	12/15 (80.0)	220/247 (89.1)
Gestational age (weeks), mean ± SD	38.6 (±2.3)	38.2 (±1.7)
Female, *n* (%)	10/15 (56.7)	129/254 (50.8)
Birth weight (grams)	3241.4 (±523.0)	3131.7 (±533.4)
Appropriate weight for gestational age, *n* (%)	14/15 (93.3)	199/247 (80.6)
Birth height (cm), mean ± SD	48.5 (±3.12)	48.2 (±2.57)
Cephalic perimeter (cm), mean ± SD	35.0 (±1.01)	34.4 (±1.98)
Mother age at delivery (years), mean ± SD	31.7 (±3.65)	34.5 (±3.19)
APGAR 1st-minute score		
Score (mean ± SD)	7.1 (±2.0)	8.0 (±1.2)
≤7	5/14 (35.7)	50/241 (20.7)
>7	9/14 (64.3)	191/241 (79.3)
APGAR 5th-minute score		
Score (mean ± SD)	8.8 (±0.70)	8.9 (±0.69)
≤7	1/14 (7.1)	10/241 (4.1)
>7	13/14 (92.9)	231/241 (95.9)
**Bayley-III Scale at 6 months of age**		
Normal, *n* (%)	5/5 (100.0)	59/95 (62.1)
Delay, *n* (%)	0/5 (0.0)	36/95 (37.9)
Cognitive, *n* (%)	0/5 (0.0)	20/36 (55.6)
Language, *n* (%)	0/5 (0.0)	23/36 (63.9)
Motor, *n* (%)	0/5 (0.0)	12/36 (33.3)
**Bayley-III Scale at 12 months of age**		
Normal, *n* (%)	3/3 (100.0)	67/100 (67.0)
Delay, *n* (%)	0/3 (0.0)	33/100 (33.0)
Cognitive, *n* (%)	0/3 (0.0)	4/33 (12.2)
Language, *n* (%)	0/3 (0.0)	26/33 (78.8)
Motor, *n* (%)	0/3 (0.0)	13/33 (39.4)
**Time of Maternal SARS-CoV-2 Infection**		
1st trimester, *n* (%)	n/a	50/254 (19.7)
2nd trimester, *n* (%)	n/a	78/254 (30.7)
3rd trimester, *n* (%)	n/a	105/254 (41.3)
At delivery, *n* (%)	n/a	21/254 (8.3)
**Severity of Maternal SARS-CoV-2 Infection**		
Non-severe, *n* (%)	n/a	212/250 (84.8)
Severe, *n* (%)	n/a	42/250 (16.8)
**Caregiver Education Level**		
No university degree, *n* (%)	11/15 (73.3)	173/243 (71.2)
University degree, *n* (%)	4/15 (26.7)	70/243 (28.8)

* n/a—not applicable.

**Table 2:** Bayley-III cognitive, language, and motor domains at 6 and 12 months after birth, according to the time of maternal SARS-CoV-2 infection.

Bayley-III Domains *		Time of Maternal SARS-CoV-2 Infection				*p-Values*
	HC	1st Trimester	2nd Trimester	3rd Trimester	At Delivery	*ANOVA or* χ^2^
**6 months after birth**						
Cognitive, mean (±SD)	110.0 (±11)	111.7 (±23)	102.0 (±12)	**98.3 (±12) 1st**	**90.0 (±10) 1st**	***0.0037***
Normal, *n* (%)	5/5 (100.0)	21/23 (91.3)	23/29 (79.3)	28/38 (73.7)	3/5 (60.0)	
Altered, *n* (%)	0/5 (0.0)	**2/23 (8.7) ^#^**	**6/29 (20.7) ^#^, 1st**	**10/38 (26.3) ^#^, 1st**	**2/5 (40.0) ^#^, 1st, 2nd, 3rd**	***0.0001***
Language, mean (±SD)	92.0 (±2)	94.4 (±17)	95.5 (±12)	93.3 (±14)	88.8 (±22)	*0.8994*
Normal, *n* (%)	5/5 (100.0)	21/23 (91.3)	23/29 (79.3)	29/38 (73.7)	3/5 (60.0)	
Altered, *n* (%)	0/5 (0.0)	**2/23 (8.7) ^#^**	**6/29 (20.7) ^#^**	**9/38 (26.3) ^#^**	**2/5 (40.0) ^#^, 1st, 2nd, 3rd**	***0.0001***
Motor, mean (±SD)	112.4 (±12)	106.6 (±21)	107.3 (±16)	104.3 (±16)	92.2 (±17)	*0.2961*
Normal, *n* (%)	5/5 (100.0)	19/23 (82.6)	26/29 (89.7)	35/38 (92.1)	3/5 (60.0)	
Altered, *n* (%)	0/5 (0.0)	**4/23 (17.4) ^#^**	**3/29 (10.3) ^#^**	**3/38 (7.9) ^#^**	**2/5 (40.0) ^#^, 1st, 2nd, 3rd**	***0.0001***
**12 months after birth**						
Cognitive, mean (±SD)	115.0 (±9)	113.8 (±15)	108.2 (±12)	110.6 (±12)	103.8 (±19)	*0.3503*
Normal, *n* (%)	3/3 (100.0)	15/15 (100.0)	33/34 (97.1)	44/45 (97.8)	4/6 (66.7)	
Altered, *n* (%)	0/3 (0.0)	0/15 (0.0)	1/34 (2.9)	1/45 (2.3)	**2/6 (33.3) ^#^, 1st, 2nd, 3rd**	***0.0001***
Language, mean (±SD)	98.0 (±5)	107.0 (±12)	92.5 (±15)	98.2 (±17)	**83.5 (±19) 1st**	***0.0161***
Normal, *n* (%)	3/3 (100.0)	15/15 (100.0)	22/34 (64.7)	34/45 (75.6)	3/6 (50.0)	
Altered, *n* (%)	0/3 (0.0)	0/15 (0.0)	**12/34 (35.3) ^#^**	**11/45 (24.4) ^#^**	**3/6 (50.0) ^#^, 1st, 2nd, 3rd**	***0.0001***
Motor, mean (±SD)	95.0 (±5)	97.3 (±13)	101.5 (±12)	103.5 (±16)	102.4 (±18)	*0.5351*
Normal, *n* (%)	3/3 (100.0)	12/15 (80.0)	31/34 (91.2)	39/45 (86.7)	5/6 (83.0)	
Altered, *n* (%)	0/3 (0.0)	**3/15 (20.0) ^#^**	**3/34 (8.8) ^#^**	**6/45 (13.3) ^#^**	**1/6 (17.0) ^#^, 2nd, 3rd**	***0.0001***

* Data are expressed as mean values (standard deviations—SDs) of Bayley-III cognitive (purple), language (green), and motor (blue) domains at 6 and 12 months after birth for infants born to mothers with SARS-CoV-2 infection during pregnancy. The *p*-values were calculated by multiple comparisons carried out by ANOVA with Bonferroni correction. The comparative analysis of the categorical data was carried out by the Chi-square test (χ^2^). Significant differences are highlighted in bold and identified by **^#^** when compared with HC and by 1st, 2nd, and 3rd when compared with the 1st trimester, 2nd trimester and 3rd trimester, respectively.

**Table 3:** Bayley-III cognitive, language, and motor domains at 6 and 12 months, according to the severity of maternal SARS-CoV-2 infection.

Bayley-III Domains *		Severity of Maternal SARS-CoV-2 Infection		*p-Values*
	HC	Non-Severe	Severe	*ANOVA or* χ^2^
**6 months after birth**				
Cognitive, mean (±SD)	110.0 (±11)	102.6 (±17)	97.9 (±15)	*0.3477*
Normal, *n* (%)	5/5 (100.0)	65/80 (81.3)	10/14 (71.4)	
Altered, *n* (%)	0/5 (0.0)	**15/80 (18.7) ^#^**	**4/14 (28.6) ^#^**	***0.0001***
Language, mean (±SD)	91.6 (±1)	94.7 (±15)	89.8 (±14)	*0.4640*
Normal, *n* (%)	5/5 (100.0)	62/80 (77.5)	9/14 (64.3)	
Altered, *n* (%)	0/5 (0.0)	**18/80 (22.5) ^#^**	**5/14 (35.7) ^#,N-S^**	***0.0001***
Motor, mean (±SD)	112.4 (±12)	106.5 (±17)	96.8 (±17)	*0.0972*
Normal, *n* (%)	5/5 (100.0)	71/80 (88.8)	11/14 (78.6)	
Altered, *n* (%)	0/5 (0.0)	**9/80 (11.2) ^#^**	**3/14 (21.4) ^#,N-S^**	***0.0001***
**12 months after birth**				
Cognitive, mean (±SD)	115.0 (±9)	109.6 (±13)	110.0 (±13)	*0.7815*
Normal, *n* (%)	3/3 (100.0)	82/85 (96.5)	11/12 (91.7)	
Altered, *n* (%)	0/3 (0.0)	**3/85 (3.5) ^#^**	**1/12 (8.3) ^#^**	***0.0095***
Language, mean (±SD)	98.0 (±5)	96.5 (±17)	100.3 (±16)	*0.7444*
Normal, *n* (%)	3/3 (100.0)	61/85 (71.8)	11/12 (91.7)	
Altered, *n* (%)	0/3 (0.0)	**24/85 (28.2) ^#^**	**1/12 (8.3) ^#,N-S^**	***0.0001***
Motor, mean (±SD)	95.0 (±5)	101.4 (±14)	107.8 (±15)	*0.2238*
Normal, *n* (%)	3/3 (100.0)	72/85 (84.7)	12/12 (100.0)	
Altered, *n* (%)	0/3 (0.0)	**13/85 (15.3) ^#^**	**0/12 (0.0) ^#,N-S^**	***0.0001***

* Data are expressed as mean values (standard deviations—SDs) of Bayley-III cognitive (purple), language (green), and motor (blue) domains at 6 and 12 months after birth for infants born to mothers with SARS-CoV-2 infection during pregnancy. The *p*-values were calculated by multiple comparisons carried out by ANOVA with Bonferroni correction. The comparative analysis of the categorical data was carried out by the Chi-square test (χ^2^). Significant differences are highlighted in bold and identified by **^#^** when compared with HC and by **^N-S^** when compared with the non-severe subgroup.

**Table 4:** Bayley-III cognitive, language, and motor domains at 6 and 12 months, according to the caregiver educational level.

Bayley-III Domains *		Caregiver Educational Level		*p-Values*
	HC	No University Degree	University Degree	*ANOVA or* χ^2^
**6 months after birth**				
Cognitive, mean (±SD)	110.0 (±11)	102.0 (±18)	103.0 (±15)	*0.5784*
Normal, *n* (%)	5/5 (100.0)	55/66 (83.3)	19/25 (76.0)	
Altered, *n* (%)	0/5 (0.0)	**11/66 (16.7) ^#^**	**6/25 (24.0) ^#^**	***0.0001***
Language, mean (±SD)	91.6 (±1)	93.3 (±13)	95.2 (±19)	*0.8032*
Normal, *n* (%)	5/5 (100.0)	50/66 (75.8)	19/25 (76.0)	
Altered, *n* (%)	0/5 (0.0)	**16/66 (24.2) ^#^**	**6/25 (24.0) ^#^**	***0.0001***
Motor, mean (±SD)	112.4 (±12)	103.6 (±17)	109.6 (±20)	*0.2356*
Normal, *n* (%)	5/5 (100.0)	57/66 (86.4)	22/25 (88.0)	
Altered, *n* (%)	0/5 (0.0)	**9/66 (13.6) ^#^**	**3/25 (12.0) ^#^**	***0.0010***
**12 months after birth**				
Cognitive, mean (±SD)	115.0 (±9)	109.9 (±13)	110.7 (±13)	*0.7926*
Normal, *n* (%)	3/3 (100.0)	63/66 (95.5)	27/28 (96.4)	
Altered, *n* (%)	0/3 (0.0)	3/66 (4.5)	1/28 (3.6)	*0.1466*
Language, mean (±SD)	98.0 (±5)	97.5 (±17)	96.6 (±15)	*0.9680*
Normal, *n* (%)	3/3 (100.0)	49/66 (74.2)	22/28 (78.6)	
Altered, *n* (%)	0/3 (0.0)	**17/66 (25.8) ^#^**	**6/28 (21.4) ^#^**	***0.0001***
Motor, mean (±SD)	95.0(±5)	102.2(±15)	103.3(±14)	*0.6285*
Normal, *n* (%)	3/3 (100.0)	57/66 (86.4)	24/28 (85.7)	
Altered, *n* (%)	0/3 (0.0)	**9/66 (13.6) ^#^**	**4/28 (14.3) ^#^**	***0.0005***

* Data are expressed as mean values (standard deviations—SDs) of Bayley-III cognitive (purple), language (green), and motor (blue) domains at 6 and 12 months after birth for infants born to mothers with SARS-CoV-2 infection during pregnancy. The *p*-values were calculated by multiple comparisons carried out by ANOVA with Bonferroni correction. The comparative analysis of the categorical data was carried out by the Chi-square test (χ^2^). Significant differences are highlighted in bold and identified by ^#^ when compared with HC.

**Table 5:** Associations between Bayley-III domains at 6 and 12 months and maternal features.

Bayley-III Domains *	Maternal Features **					
	Acute SARS-CoV-2 Infection at Delivery	*p-Value*	Severe Disease	*p-Value*	University Degree	*p-Value*
**6 months after birth**						
Cognitive, Coef (SE)	−12.33 (7.58)	*0.107*	−4.71 (4.82)	*0.332*	1.03 (3.95)	*0.795*
Language, Coef (SE)	−6.86 (6.63)	*0.304*	−4.89 (4.19)	*0.247*	1.92 (3.45)	*0.579*
Motor, Coef (SE)	−15.80 (7.83)	*0.047*	−9.68 (4.97)	*0.055*	5.94 (4.12)	*0.153*
**12 months after birth**						
Cognitive, Coef (SE)	**−14.65 (5.30)**	***0.007***	0.41 (4.08)	*0.920*	0.79 (2.98)	*0.792*
Language, Coef (SE)	**−14.01 (6.76)**	***0.041***	3.83 (5.07)	*0.452*	−0.88 (3.67)	*0.812*
Motor, Coef (SE)	0.12 (5.89)	*0.983*	6.40 (4.29)	*0.140*	1.07 (3.21)	*0.740*

* Multinomial and logistic regression models were employed to examine the associations between Bayley-III scores (cognitive—purple; language—green; motor—blue) and maternal features. The regression models were adjusted for infant sex and gestational age at delivery. ** Reference groups: infections in the 1st, 2nd, and 3rd trimesters; non-severe; and no-university degree. *p*-Values of <0.05 denoted statistical significance. The analyses were performed with Minitab version 17 (Minitab, LLC, State College, PA, USA). Significant coefficients are highlighted in bold.

## Data Availability

No new data were created or made available due to privacy and ethical restrictions on pregnant woman and pediatric data. Therefore, the data cannot be shared to ensure the confidentiality and protection of the participants’ privacy.
